# A Case in Practice

**Published:** 1883-12

**Authors:** W. P. Richards

**Affiliations:** Orange, N. J.


### A CASE IN PRACTICE.

BY DR. W. P. RICHARDS, ORANGE, N. J.

READ BEFORE THE CENTRAL DENTAL ASSOCIATION OF NEW JERSEY.

The subject I desire to present this evening is Ulceration of the Antrum, and my process of treatment. To some of you it may be known, but I have never heard this method spoken of. I will mention a case, in order that I may be better understood.

About three years ago a lady suffering from this disease came to me for advice. I asked her to describe the symptoms, which she briefly did, as follows: "I have no appetite, have dull pains in the head, and my husband often speaks to me about my offensive breath."

Upon making an examination I found necrosed the left upper central and lateral incisors, also the canine, from which originated the trouble. I asked her how it happened there were so many dead teeth in her mouth. She informed me that while on a visit near Newton (in this State) she had trouble with her teeth,and consulted a dentist in that place. His method of procedure seemed to be to apply that much-abused remedy, arsenic, in all the teeth mentioned, then dismissing her without making further appointments, that he might remove the devitalized pulps and commence a proper course of treatment.

Over the canine was a slight opening discharging pus. Probing carefully, I found that the instrument extended along the palatine arch until the patient informed me that it could be felt with the tongue. I then noticed in the vault of the mouth a dark red spot, about the size of a pea, but did nothing to it at that visit, and only syringed out the opening on the bucal surface with carbolic acid and warm water. I also drilled out the fang of the canine tooth from the lingual surface, in order to give vent to the accumulated pus, requesting the patient to call next day. Upon returning she complained that the roof of her mouth was very sore to the touch. The dark spot had become somewhat larger and quite soft. I came to the conclusion that an opening was necessary. Using the lance, a large quantity of pus followed. The weather was cold enough to have windows closed, but it did not take long to open them and let in plenty of fresh air, which was necessary because of the terribly fetid odor of the liberated pus. The antrum was freely washed out with permanganate of potash and water.

After the patient had left I contrived the rubber drainage tube seen in the model now before you. To insert this tube I used a pair of long slender pliers, and was careful to see that the silver band at the end of the tube fitted snugly around the opening.

There was now a clear unobstructed channel to the seat of war, and no danger of its closing up to retain pus. This tube was worn without discomfort, is easily made, saves time, trouble, and a cure can be effected sooner, I believe, than by any other method. Should you ever have such a case it is well worth a trial.

The essential feature in the treatment of this case was the insertion into the opening in the palatine arch of a silver canula, with a broad flange, sufficient to prevent its passing entirely into the antrum. To this was attached a rubber drainage tube, which could be utilized as well in the injection of the proper remedies.—EDITOR.

